# Surgical Treatment of Atrial Septal Defects

**DOI:** 10.31083/j.rcm2510350

**Published:** 2024-09-29

**Authors:** Philippe Grieshaber, Christoph Jaschinski, Mina Farag, Elizabeth Fonseca-Escalante, Matthias Gorenflo, Matthias Karck, Tsvetomir Loukanov

**Affiliations:** ^1^Division of Congenital Cardiac Surgery, Department of Cardiac Surgery, University Hospital Heidelberg, 69120 Heidelberg, Germany; ^2^Department of Pediatric Cardiology and Congenital Heart Disease, University Hospital Heidelberg, 69120 Heidelberg, Germany; ^3^Department of Cardiac Surgery, University Hospital Heidelberg, 69120 Heidelberg, Germany

**Keywords:** atrial septal defect, surgery, congenital heart disease, operative technique

## Abstract

Atrial septal defects (ASDs) are among the most prevalent congenital cardiac malformations. Closure of the defect and repair of associated cardiac malformations are typically indicated if an ASD is hemodynamically significant or symptomatic. This narrative review aims to summarize key aspects of surgical ASD closures. A non-systematic literature review was conducted to cover surgically relevant aspects of (developmental) anatomy, morphology, and treatment. ASDs result from diverse developmental alterations, leading to subtype-specific associated cardiac malformations, meaning surgical therapy varies accordingly. Presently, surgical repair yields excellent outcomes for all ASD subtypes, with minimally invasive approaches, especially in adults, increasingly employed for ASD closure. Surgical ASD repair is safe with excellent results. However, familiarity with ASD subtypes and typically associated lesions is crucial for optimal patient management.

## 1. Introduction

Atrial septal defects (ASDs), defined as communications between the two atria of the heart, can occur alone or in conjunction with other congenital malformations. ASDs rank as the second-most common congenital cardiac malformation, occurring in 1.65/1000 live births and constituting 10 to 15% of all congenital cardiac malformations [1]. ASDs encompass various lesions with different embryological origins [2]. Meanwhile, these differences impact the decision-making regarding treatment indications and modalities and are reviewed in the following section. Pathophysiologically, ASDs in an otherwise normal four-chambered heart can lead to a shunt on the atrial level between the left atrium that receives the pulmonary venous return and the right atrium that receives the systemic venous return. The shunt volume is determined by the morphology and size of the ASD, by the pressure difference between the left and right atria resulting from right ventricular compliance and pulmonary vascular resistance (PVR). The shunt is usually directed from the left to the right atrium, resulting in a volume load of the right heart (left-to-right shunt). Contrarily to ventricular septal defects, ASDs do not impose pressure load on the right ventricle [3]. The shunt volume is usually described as the ratio of pulmonary blood flow (Q_p_) and systemic blood flow (Q_s_), with a Q_p_/Q_s_of more than 1.5 considered hemodynamically relevant in children and adults with ASD [4, 5, 6]. Symptoms are variable and usually less pronounced compared with other shunt lesions. Consecutively, ASDs are detected at a wide variety of ages, with symptoms typically resulting from pulmonary overcirculation and right heart failure (intolerance to exercise, failure to thrive) or (particularly in adults) from thromboembolic events caused by paradoxical embolism [7]. The natural history of untreated ASD, known from historical data, is associated with right heart failure and an increased annualized mortality risk beginning from young adulthood, resulting in a markedly reduced median life expectancy. However, given the variable clinical courses, some patients remain symptom-free until advanced adulthood [8, 9]. A landmark paper by Murphy *et al*. [10] showed that the life expectancy of ASD patients remains normal if surgical treatment is undertaken before the age of 25. Contrarily, if treated at an older age, patients exhibit increased mortality rates compared to the average population [10]. Therefore, surgical closure of ASDs has become universally accepted as the treatment of choice for patients with hemodynamically relevant ASD or symptoms related to ASD. Additionally, interventional closure techniques using closure devices have exceptionally evolved, offering now valid therapeutical strategies for certain ASD subtypes [11, 12, 13]. This review aims to focus on the surgical treatment of ASD, summarizing the current state of surgical techniques for different ASD subtypes and the choice of the surgical approach. Furthermore, the results, complications, and impact on the prognosis of surgical ASD treatment are discussed.

## 2. Materials and Methods

A narrative, non-systematic review was conducted, defining subjects to be covered in advance. Literature searches were performed in PubMed using appropriate search and MeSH (Medical Subject Headings) terms. Additionally, potentially relevant literature not listed in PubMed was extracted from Google Scholar. The search was conducted without time limits, and the following search terms were used in various combinations: ‘atrial septal defect’, ‘ASD’, ‘surgical treatment’, ‘embryology’, ‘anatomy’, and ‘morphology’. Original works, case reports, case series, and reviews were considered eligible literature sources for this review.

## 3. Results

### 3.1 Developmental Anatomy of the Interatrial Septum and ASD Subtypes

A comprehensive understanding of the term ‘ASD’ goes beyond the notion of a simple hole in the interatrial septum. A deeper understanding of the anatomy of the interatrial septum, the resulting subtypes of ASD, and particularly the typically associated subtype-specific malformations, is crucial for developing tailored surgical treatment strategies and ensuring patient safety.

The relevant processes of the formation of the atria and the interatrial septum occur between the fourth and sixth weeks of gestation. After simplifying these complex processes for this review, three significant aspects emerged:

Firstly, a common atrium forms from the inlet portion of the heart tube around the fourth week of gestation. This atrium receives blood from two venous channels, which develop into the two horns of the sinus venosus. Eventually, this sinus venosus then septates into the coronary sinus and the two venae cavae. An additional venous channel will connect posteriorly to the sinus venosus, forming the pulmonary veins. These veins then migrate left alongside the posterior wall of the sinus venosus. Disruptions in the septation of the evolving coronary sinus from the atrium will result in a communication between the coronary sinus and the left atrium, namely a coronary sinus ASD. These rare defects account for less than 1% of all ASDs [14].

Anomalies in the leftward migration of the pulmonary veins will result in an abnormally right-sided and anterior position of the right pulmonary veins. This anterior position can lead to a loss of septation between the coronary sinus and, in most cases, the upper cavoatrial junctions. This leads to superior sinus venosus ASD, which represents 4 to 11% of all ASDs, whereas inferior sinus venosus ASD is much rarer, accounting for less than 1% of all ASDs [15].

Secondly, the common atrium undergoes septation into the left and right atria. A septum primum grows from the atrial roof towards the endocardial cushions, forming the atrioventricular (AV) junction. The space between the growing septum primum and the AV junction is the ostium primum, which is gradually obliterated as the septum primum grows. To facilitate blood flow into the left heart, the septum primum perforates subsequently in its cranial aspect, forming the ostium secundum. Concurrently, an inward fold in the atrial roof to the right of the septum primum develops. This fold (septum secundum) overlaps with the ostium secundum, creating a flap valve and enabling right-to-left-shunting. The caudal end of the septum secundum forms the limbus of the fossa ovalis. The region caudal to the limbus is called the fossa ovalis. Postnatally, as the pressure in the left atrium exceeds the right atrial pressure, the septum primum is pressed against the septum secundum, and the flap valve closes. Subsequently, the tissue of the septum primum and the septum secundum merge. If the septum secundum insufficiently overlaps the ostium secundum, a tissue defect in the septum primum at the fossa ovalis, the secundum ASD, will remain. Secundum ASDs are the most common ASD subtype and constitute about 75% of all ASDs [16]. If there is no tissue deficiency but a lack of flap valve–tissue merging, a patent foramen ovale (PFO) consequently forms; PFOs exist in about 25 to 30% of the general population [17].

Thirdly, the AV canal undergoes septation into left- and right-sided AV valves through ingrowth and fusion of a superior and an inferior endocardial cushion. Additionally, two lateral endocardial cushions grow towards the center of the AV canal, contributing to the AV valve formation [18]. The septum primum joins the crest formed by the fused inferior and superior endocardial cushions, leading to the closure of the atrial septum at this site. If this fusion is incomplete, i.e., the foramen primum remains patent, a primum ASD is formed [19], which accounts for about 10% of all ASDs [20].

### 3.2 Indications for Surgical ASD Therapy

Closure of an ASD may be warranted for several reasons: hemodynamic relevance of the shunt (Q_p_/Q_s_>1.5, echocardiographic signs of right heart volume overload), presence of heart failure symptoms related to the ASD (or its associated lesions), or the occurrence of paradoxical embolism. In cases where PVR is elevated, the risk of right heart failure after ASD closure needs consideration. A PVR exceeding 8 Wood units × m^2^ without vasoreagibility is a contra-indication for ASD closure. In less pronounced cases, a fenestrated ASD closure combined with medical pulmonary hypertension treatment may be an option [21, 22].

In recent years, interventional treatment options have grown safer and more effective, meaning that for a subset of ASDs (namely PFOs and secumdum ASDs), interventional closure has become an accepted alternative to surgical treatment for eligible patients [23]. Transcatheter closure of sinus venosus ASDs using covered stents has been reported to be feasible; however, it is still associated with high rates of major complications, such as stent embolization and caval vein occlusion [24].

### 3.3 Timing of Surgery

In children, ASD closure is typically scheduled electively. Spontaneous closure of the ASD, mostly seen in small secundum defects or PFOs, is unlikely beyond three years [25]. Therefore, surgical ASD closure in asymptomatic children is often performed conveniently between the ages of three and school age. However, intolerance to exercise or large shunt volumes with severe right heart enlargement might warrant earlier closure. Infants presenting with severe heart failure symptoms alongside an isolated ASD should prompt suspicion of additional underlying conditions. In adults, unnecessarily delaying ASD closure should be avoided, as this might decrease the likelihood of normalizing right heart function, increase the risk of arrhythmias, and also potentially reduce life expectancy [10, 26, 27].

### 3.4 Specific Considerations and Surgical Results for ASD Subtypes

#### 3.4.1 General Considerations

For all ASD subtypes, a thorough echocardiographic evaluation should be conducted both preoperatively and intraoperatively to rule out the presence of more than one lesion in the atrial septum (such as an additional sinus venosus ASD in a patient scheduled for PFO closure) [28]. Surgical ASD closure necessitates the use of cardiopulmonary bypass with bicaval canulation to obtain a bloodless operative field. During intracardiac correction, primarily performed via a right atriotomy, it is crucial to avoid air embolism into the ascending aorta. While some surgeons consider induced ventricular fibrillation adequate for the relatively brief period of intracardiac surgery, we advocate for the safer approach of aortic clamping and cardioplegia application. Upon opening the right atrium, meticulous anatomical inspection is imperative to exclude concomitant ASDs and anomalously returning pulmonary veins. Furthermore, it is essential to accurately locate the coronary sinus, the triangle of Koch, and the openings of the superior and inferior venae cavae. Following correction, residual shunts should be excluded using echocardiography with or without the Valsalva maneuver and bubble testing [29].

#### 3.4.2 Secundum ASD, PFO

Surgical closure of a PFO as an isolated lesion has become exceedingly rare due to the advancements in interventional PFO closure [30]. Larger secundum ASDs are frequently not suitable for percutaneous closure and are thus referred for surgical correction. Intraoperatively, the initial inspection should determine the size of the defect. The border zone between the limbus of the fossa ovalis and the septum primum should be carefully inspected for additional fenestrations. If multiple fenestrations are identified, resectioning the septum primum tissue may be reasonable. Moreover, the inferior border of the ASD should be well-defined. A decision must be made regarding whether to close the ASD with or without patch material. Notably, a PFO can frequently be closed with direct continuous or interrupted sutures, obviating the need for patch material [31]. However, for larger secundum ASDs, utilizing patch material (e.g., autologous pericardium fixed in formaldehyde or xenogeneic patch material) offers a safe means of preventing tears and residual defects [32]. The patch is typically secured to the ASD borders using continuous non-resorbable sutures. Our group previously described the operative technique in a video tutorial [33].

Contemporary studies on surgical closure of secundum ASD report low perioperative mortality rates of 0 to 0.3% [34, 35]. However, preoperative severe tricuspid regurgitation and severe right heart dysfunction appear to diminish long-term survival. Postoperatively, pericardial effusions develop in about 10 to 15% of patients within up to two months after surgery, underscoring the need for regular echocardiographic monitoring [36].

#### 3.4.3 Primum ASD

Primum ASD represents an endocardial cushion defect and, therefore, is often accompanied by additional lesions. A cleft in the left AV valve between the inferior and superior bridging leaflet is present in 100% of cases. Additional left-sided malformations, such as single papillary muscle or left-ventricular outflow tract (LVOT) obstruction, are present in approximately 10% of cases [37]. The AV valves can be separated (with two distinct valve annuli) or configured as a common valve. In the latter scenario, the AV node is displaced posteriorly and lies between the coronary sinus and the inferior border of the AV valve—surgical correction aims to close the defect and repair the left AV valve. Closure of the ASD with a patch follows a similar approach to that described for secundum ASD. However, one has to consider that the sutures of the caudal border of the ASD are placed directly at the AV valve plane. Depending on the presence of a small rim in this region and the tissue quality, the patch can be affixed using continuous sutures with superficial stitches or interrupted sutures placed from the right ventricular side through the AV valve plane and the patch [20, 37, 38]. Different techniques exist in the AV node region to prevent AV block [39]. While some surgeons avoid this region entirely by suturing the patch around the coronary sinus and allowing the coronary sinus to drain to the left-atrial side, we believe the risk of AV block can be sufficiently mitigated by placing stitches superficially in this region and staying close to the AV valve annulus.

While perioperative mortality rates after primum ASD repair are low, a notable risk for re-operations primarily arises from left AV valve dysfunction. Indeed, re-operations between 5 and 15% have previously been reported [40, 41, 42]. Another cause to re-do surgery in these patients is the development of LVOT obstruction due to the anatomically frequently narrow and long LVOT, with additional fibrous obstruction developing over time [43, 44, 45].

#### 3.4.4 Upper and Lower Sinus Venosus ASD

The upper sinus venosus defect is usually located near the upper cavo-atrial junction. The right upper pulmonary veins may either drain into the region of the ASD in the left atrium (functional partial anomalous pulmonary vein return (PAPVR)) or drain directly into the right atrium or the superior vena cava (anatomical PAPVR). Preoperative diagnostics should identify the presence of PAPVR and the anatomical locations of anomalously returning pulmonary veins. Surgical correction aims to close the ASD and redirect pulmonary venous return to the left atrium. Therefore, the patch used for ASD closure is implanted to retain the pulmonary veins on the left atrial side of the patch. The higher the pulmonary veins return to the superior vena cava (SVC), the longer this tunnel must be. A common problem with tunneling PAPVR to the SVC is obstruction of either the pulmonary venous tunnel or the SVC. To prevent SVC stenosis, a second patch can be used to augment the cavoatrial junction and the SVC (two-patch technique) [46, 47]. However, this technique, which involves an incision in the cavoatrial junction, has been associated with higher rates of postoperative sinus node dysfunction than the single-patch technique [48, 49, 50]. The Warden procedure is a technique to correct particularly remote PAPVR into the SVC [51]. This technique divides the SVC cranially into the uppermost pulmonary vein and closes the stump. The ASD patch is sutured so the anomalous pulmonary vein completely drains to the left atrium, and the cranial stump of the SVC is anastomosed to the right atrial appendage. While this technique avoids incisions in the cavoatrial junction, it might be associated with higher rates of SVC obstructions, particularly in the region of the cavoatrial anastomosis. A wide and tension-free anastomosis should be fashioned to prevent this after carefully removing all trabeculations inside the right atrial appendage [52, 53, 54, 55]. In adults, modifications of the Warden technique using interposition grafts between the SVC and the right atrium have been described [56, 57, 58].

Lower sinus venosus defects are rare, often near the inferior cavoatrial junction. The right lower pulmonary veins can frequently connect to the right atrium or even the inferior vena cava (IVC) in this area. Surgical correction follows the same principles described for the upper sinus venosus defects, aiming to close the ASD while re-routing the pulmonary venous return to the left atrium. To facilitate the exposure of the ASD and the pulmonary veins, the IVC should be cannulated as far caudally as possible. In larger patients, a femoral venous cannula may enhance exposure.

The perioperative mortality is similarly low to that described for other ASD subtypes. The main long-term issues result from sinus node dysfunction and stenoses of the SVC and pulmonary venous baffles. The Warden procedure has been described as reducing sinus node dysfunction compared with the two-patch technique [48, 59]. Meanwhile, SVC obstruction after the Warden procedure has been described to occur in 2 to 5% of patients [60, 61].

#### 3.4.5 Coronary Sinus ASD

In this exceedingly rare ASD subtype, there is a tissue deficiency between the coronary sinus and the left atrium. It is also referred to as ‘unroofed coronary sinus’. The extent of this deficiency can vary from one or more small defects to a complete absence of a roof over the coronary sinus. The lesion is frequently associated with a persisting left-sided superior vena cava (LSVC), which typically drains into the unroofed coronary sinus or, if the deficiency extends over the entire coronary sinus, into the left atrial roof. Various surgical approaches exist to correct this lesion, aiming to eliminate the interatrial communication and re-route the LSVC to the right atrium [62]. In this regard, a roof of the coronary sinus, which also tunnels the LSVC via the coronary sinus to the right atrium, is constructed [63]. If this approach is used, care must be taken to avoid compromising the pulmonary venous return or the mitral valve inflow. If a neo-roof of the coronary sinus cannot be constructed with reasonable risk, the opening of the coronary sinus to the right atrium can be closed with a patch, while the LSVC is transected and anastomosed to the right atrial appendage, either directly or with an interposition graft. This leaves the coronary sinus draining into the left atrium, which usually does not cause detectable desaturation.

Long-term data on outcomes for this rare ASD subtype are limited. However, current case reports and small series suggest favorable long-term results following surgical repair [64, 65].

#### 3.4.6 Surgical Correction after Previous Percutaneous ASD Closure

In certain cases, surgical ASD closure may be necessary following previous percutaneous device closure. Indications for this approach include device endocarditis, device malposition, incomplete device closure, and the presence of an additional, frequent superior sinus venosus defect, which has not been detected before the device closure of the concomitant PFO [66]. If device endocarditis is present, the device should be completely explanted during surgery, and the resulting defect should preferably be closed with an autologous patch [67, 68]. In the other previously mentioned scenarios, the grade of device ingrowth and the associated risks of device explanation determine whether the device is left in place while closing the additional shunt.

A more far-reaching indication for surgery arises from the perforation of a closure device, which can affect the aortic root or the atrial roof [69]. The resulting hemopericardium frequently leads to rapid circulatory deterioration. If the patients reach the hospital, emergent sternotomy and connection to the heart–lung machine are undertaken to stabilize the patient. Following induction of cardioplegic arrest, the situation can be assessed. The surgical correction should include explantation of the device and perforation repair. This can be achieved by reconstructing the atrial roof and the interatrial septum with patches or, in the case of aortic root perforation, by an aortic root replacement [70, 71].

### 3.5 Surgical Access

While conventional access for surgical ASD closure has been a standard median sternotomy, different alternative access routes have recently been suggested that aim at being minimally invasive and reducing surgical trauma.

These alternative approaches include lower hemi-sternotomy, right lateral thoracotomy, and, particularly for larger patients, totally endoscopic approaches via right lateral mini-thoracotomy. For right lateral thoracotomy, both horizontal (parallel to the ribs) and vertical (craniocaudal) skin incisions have been described. The vertical skin incisions appear to provide a better exposure of the situs than the horizontal approach [72, 73, 74, 75, 76]. Generally, in minimally invasive approaches, the surgeon must decide whether central cannulation or peripheral cannulation is preferred. Peripheral cannulation offers greater access to the surgical field. However, the risk of ischemic complications after femoral cannulation needs to be considered, especially for smaller patients and longer cardiopulmonary bypass durations.

For adults, the totally endoscopic approach presents an elegant solution. Cardiopulmonary bypass is established via the groin vessels. An additional venous cannula in the SVC inserted via the internal jugular vein offers, from our experience, a comfortable way to conduct total bypass without interfering with the operative situs. Results of totally endoscopic ASD closure, primarily in secundum ASDs, show that the cardiopulmonary bypass and cardioplegic arrest times are longer than with the median sternotomy approach. However, the total duration of surgery does not appear substantially longer, and recovery times may even be shorter than after conventional ASD closure [77, 78, 79, 80].

From our perspective, each program should define its preferred pathway to achieve the best possible surgical results, prioritizing maximal patient safety. If minimally invasive approaches do not compromise these aspects, they should be considered equivalent or even superior (owing to cosmetic advantages, the avoidance of sternotomy, and possibly accelerated recovery) to the standard approach via a median sternotomy. To enhance process safety, minimizing inter-surgeon variability within a program is crucial.

## 4. Discussion

This non-systematic review narrative aims to provide an overview of the relevant points surgeons should be familiar with when treating patients with different ASD subtypes. As demonstrated, ASDs encompass a wide spectrum of defect morphologies and subtypes. While the majority of ASDs consist of secundum ASDs and PFOs, other subtypes pose specific morphological particularities and associated malformations. Thus, surgeons must know these nuances to address these challenges effectively. Additionally, this underscores the importance of precise preoperative diagnostics and multidisciplinary planning of the appropriate approach within a dedicated heart team with expertise in treating congenital heart defects.

## 5. Conclusions

Surgical treatment of ASD is safe and effective, with low rates of severe complications and good predictability and reproducibility of results. It is key for the treating surgeon to be familiar with the different anatomical atrial septal defect subtypes and the typically associated lesions to treat the patient with maximized safety. The surgical treatment of ASD generally allows for normalization of cardiac function and life expectancy.

## References

[1] van der Linde D, Konings EEM, Slager MA, Witsenburg M, Helbing WA, Takkenberg JJM (2011). Birth prevalence of congenital heart disease worldwide: a systematic review and meta-analysis. *Journal of the American College of Cardiology*.

[2] Briggs LE, Kakarla J, Wessels A (2012). The pathogenesis of atrial and atrioventricular septal defects with special emphasis on the role of the dorsal mesenchymal protrusion. *Differentiation; Research in Biological Diversity*.

[3] Le Gloan L, Legendre A, Iserin L, Ladouceur M (2018). Pathophysiology and natural history of atrial septal defect. *Journal of Thoracic Disease*.

[4] Baumgartner H, De Backer J, Babu-Narayan SV, Budts W, Chessa M, Diller GP (2021). 2020 ESC Guidelines for the management of adult congenital heart disease: The Task Force for the management of adult congenital heart disease of the European Society of Cardiology (ESC). Endorsed by: Association for European Paediatric and Congenital Cardiology (AEPC), International Society for Adult Congenital Heart Disease (ISACHD). *European Heart Journal*.

[5] Egidy Assenza G, Krieger EV, Baumgartner H, Cupido B, Dimopoulos K, Louis C (2021). AHA/ACC vs ESC Guidelines for Management of Adults With Congenital Heart Disease: JACC Guideline Comparison. *Journal of the American College of Cardiology*.

[6] Driscoll D, Allen HD, Atkins DL, Brenner J, Dunnigan A, Franklin W (1994). Guidelines for evaluation and management of common congenital cardiac problems in infants, children, and adolescents. A statement for healthcare professionals from the Committee on Congenital Cardiac Defects of the Council on Cardiovascular Disease in the Young, American Heart Association. *Circulation*.

[7] Farjat-Pasos JI, Chamorro A, Lanthier S, Robichaud M, Mengi S, Houde C (2023). Cerebrovascular Events in Older Patients With Patent Foramen Ovale: Current Status and Future Perspectives. *Journal of Stroke*.

[8] Campbell M (1970). Natural history of atrial septal defect. *British Heart Journal*.

[9] Doty DB, Wright CB, Hiratzka LF, Eastham CL, Marcus ML (1984). Coronary reserve in volume-induced right ventricular hypertrophy from atrial septal defect. *The American Journal of Cardiology*.

[10] Murphy JG, Gersh BJ, McGoon MD, Mair DD, Porter CJ, Ilstrup DM (1990). Long-term outcome after surgical repair of isolated atrial septal defect. Follow-up at 27 to 32 years. *The New England Journal of Medicine*.

[11] Goh E, Mohammed H, Salmasi MY, Ho S, Benedetto U, Caputo M (2022). Minimally invasive versus transcatheter closure of secundum atrial septal defects: a systematic review and meta-analysis. *Perfusion*.

[12] Baroutidou A, Arvanitaki A, Farmakis IT, Patsiou V, Giannopoulos A, Efthimiadis G (2023). Transcatheter closure of atrial septal defect in the elderly: a systematic review and meta-analysis. *Heart (British Cardiac Society)*.

[13] Chambault AL, Olsen K, Brown LJ, Mellor SL, Sorathia N, Thomas AE (2022). Transcatheter versus surgical closure of atrial septal defects: a systematic review and meta-analysis of clinical outcomes. *Cardiology in the Young*.

[14] Babapoor-Farrokhran S, Kalla A, Bozorgnia B, Amanullah A (2020). Wide unroofed coronary sinus and cryptogenic stroke: a case report. *European Heart Journal. Case Reports*.

[15] Attenhofer Jost CH, Connolly HM, Danielson GK, Bailey KR, Schaff HV, Shen WK (2005). Sinus venosus atrial septal defect: long-term postoperative outcome for 115 patients. *Circulation*.

[16] Kuijpers JM, Mulder BJM, Bouma BJ (2015). Secundum atrial septal defect in adults: a practical review and recent developments. *Netherlands Heart Journal: Monthly Journal of the Netherlands Society of Cardiology and the Netherlands Heart Foundation*.

[17] Koutroulou I, Tsivgoulis G, Tsalikakis D, Karacostas D, Grigoriadis N, Karapanayiotides T (2020). Epidemiology of Patent Foramen Ovale in General Population and in Stroke Patients: A Narrative Review. *Frontiers in Neurology*.

[18] Person AD, Klewer SE, Runyan RB (2005). Cell biology of cardiac cushion development. *International Review of Cytology*.

[19] Naqvi N, McCarthy KP, Ho SY (2018). Anatomy of the atrial septum and interatrial communications. *Journal of Thoracic Disease*.

[20] Losay J, Rosenthal A, Castaneda AR, Bernhard WH, Nadas AS (1978). Repair of atrial septal defect primum. Results, course, and prognosis. *The Journal of Thoracic and Cardiovascular Surgery*.

[21] Fraisse A, Latchman M, Sharma SR, Bayburt S, Amedro P, di Salvo G (2018). Atrial septal defect closure: indications and contra-indications. *Journal of Thoracic Disease*.

[22] Gorenflo M, Ziesenitz VC (2021). Treatment of pulmonary arterial hypertension in children. *Cardiovascular Diagnosis and Therapy*.

[23] (2017). Guidelines for the Management of Congenital Heart Diseases in Childhood and Adolescence. *Cardiology in the Young*.

[24] Rosenthal E, Qureshi SA, Jones M, Butera G, Sivakumar K, Boudjemline Y (2021). Correction of sinus venosus atrial septal defects with the 10 zig covered Cheatham-platinum stent - An international registry. *Catheterization and Cardiovascular Interventions: Official Journal of the Society for Cardiac Angiography & Interventions*.

[25] Wang SY, Welch TD, Elfenbein A, Kaplan AV (2018). Spontaneous Closure of a Secundum Atrial Septal Defect. *Methodist DeBakey Cardiovascular Journal*.

[26] Hörer J, Müller S, Schreiber C, Kostolny M, Cleuziou J, Prodan Z (2007). Surgical closure of atrial septal defect in patients older than 30 years: risk factors for late death from arrhythmia or heart failure. *The Thoracic and Cardiovascular Surgeon*.

[27] Min SY, Lee JS, Park DW, Song JM (2022). Clinical outcomes of early closure versus conservative strategy after diagnosis with atrial septal defect: a nationwide population-based cohort study. *European Journal of Cardio-thoracic Surgery: Official Journal of the European Association for Cardio-thoracic Surgery*.

[28] Rao PS (2022). Role of Echocardiography in the Diagnosis and Interventional Management of Atrial Septal Defects. *Diagnostics (Basel, Switzerland)*.

[29] Greim CA, Trautner H, Krämer K, Zimmermann P, Apfel CC, Roewer N (2001). The detection of interatrial flow patency in awake and anesthetized patients: a comparative study using transnasal transesophageal echocardiography. *Anesthesia and Analgesia*.

[30] Sperlongano S, Giordano M, Ciccarelli G, Bassi G, Malvezzi Caracciolo D’Aquino M, Del Giudice C (2022). Advances in Percutaneous Patent Foramen Ovale Closure: From the Procedure to the Echocardiographic Guidance. *Journal of Clinical Medicine*.

[31] Liava’a M, Kalfa D (2018). Surgical closure of atrial septal defects. *Journal of Thoracic Disease*.

[32] Satsangi A, Singh B, Puri S (2020). Operative Steps for OS-ASD Closure for Beginners. *IJCTS*.

[33] Jaschinski C, Loukanov T (2017). Atriumseptumdefekt. *Z Herz- Thorax- Gefäßchir*.

[34] Kim MH, Shim YH, Kim MS, Shin YS, Lee HJ, Lee JS (2017). The efficacy of elastomeric patient-control module when connected to a balloon pump for postoperative epidural analgesia: A randomized, noninferiority trial. *Medicine*.

[35] Dhaliwal R, Bhat D, Puri D, Sidhu K, Rana S, Thingam S (2000). Results of surgical closure of isolated secundum atrial septal defect. *Indian Journal of Thoracic and Cardiovascular Surgery*.

[36] Jones DA, Radford DJ, Pohlner PG (2001). Outcome following surgical closure of secundum atrial septal defect. *Journal of Paediatrics and Child Health*.

[37] Manning PB (2004). Repair of primum ASD with cleft mitral valve. *Operative Techniques in Thoracic and Cardiovascular Surgery*.

[38] Waqar T, Riaz MU, Shuaib M (2017). Surgical repair of partial atrioventricular septal defect. *Pakistan Journal of Medical Sciences*.

[39] Koshiyama H, Ishidou M, Ito H, Hirose K, Sakamoto K, Ikai A (2023). Simplified primum ASD closure technique in complete VSD repair: shallow suture crossing on the AV node. *General Thoracic and Cardiovascular Surgery*.

[40] Chowdhury UK, Airan B, Malhotra A, Bisoi AK, Kalaivani M, Govindappa RM (2009). Specific issues after surgical repair of partial atrioventricular septal defect: actuarial survival, freedom from reoperation, fate of the left atrioventricular valve, prevalence of left ventricular outflow tract obstruction, and other events. *The Journal of Thoracic and Cardiovascular Surgery*.

[41] Pontailler M, Demondion P, Lebreton G, Golmard JL, Leprince P (2017). Experience with Extracorporeal Life Support for Cardiogenic Shock in the Older Population more than 70 Years of Age. *ASAIO Journal (American Society for Artificial Internal Organs: 1992)*.

[42] Meijboom EJ, Ebels T, Anderson RH, Schasfoort-van Leeuwen MJ, Deanfield JE, Eijgelaar A (1986). Left atrioventricular valve after surgical repair in atrioventricular septal defect with separate valve orifices (“ostium primum atrial septal defect”): an echo-Doppler study. *The American Journal of Cardiology*.

[43] Perez Y, Dearani JA, Miranda WR, Stephens EH (2023). Subaortic Stenosis in Adult Patients With Atrioventricular Septal Defect. *The Annals of Thoracic Surgery*.

[44] Ivanov Y, Buratto E, Naimo P, Lui A, Hu T, d’Udekem Y (2022). Incidence and management of the left ventricular outflow obstruction in patients with atrioventricular septal defects. *Interactive Cardiovascular and Thoracic Surgery*.

[45] Gurbuz AT, Novick WM, Pierce CA, Watson DC (1999). Left ventricular outflow tract obstruction after partial atrioventricular septal defect repair. *The Annals of Thoracic Surgery*.

[46] Duncan BW (2006). Sinus Venosus Atrial Septal Defect: Repair with an Intra-Superior Vena Cava Baffle. *Operative Techniques in Thoracic and Cardiovascular Surgery*.

[47] Iyer AP, Somanrema K, Pathak S, Manjunath PY, Pradhan S, Krishnan S (2007). Comparative study of single- and double-patch techniques for sinus venosus atrial septal defect with partial anomalous pulmonary venous connection. *The Journal of Thoracic and Cardiovascular Surgery*.

[48] Jaschinski C, Cussigh C, Fonseca E, Bruckner T, Karck M, Loukanov T (2020). A Wide Comparison of Techniques for Repair of PAPVCs: One Institution’s 20-Year Experience. *The Thoracic and Cardiovascular Surgeon*.

[49] Said SM, Burkhart HM, Schaff HV, Cetta F, Phillips SD, Barnes RD (2012). Single-patch, 2-patch, and caval division techniques for repair of partial anomalous pulmonary venous connections: does it matter?. *The Journal of Thoracic and Cardiovascular Surgery*.

[50] Pace Napoleone C, Mariucci E, Angeli E, Oppido G, Gargiulo GD (2014). Sinus node dysfunction after partial anomalous pulmonary venous connection repair. *The Journal of Thoracic and Cardiovascular Surgery*.

[51] Gustafson RA (2006). Cavo-Atrial Anastomosis Technique for Partial Anomalous Pulmonary Venous Connection to the Superior Vena Cava-The Warden Procedure. *Operative Techniques in Thoracic and Cardiovascular Surgery*.

[52] Binsalamah ZM, Ibarra C, Edmunds EE, Qureshi AM, Adachi I, Caldarone CA (2021). Younger Age at Operation Is Associated With Reinterventions After the Warden Procedure. *The Annals of Thoracic Surgery*.

[53] Griffeth EM, Dearani JA, Mathew J, Graham GC, Connolly HM, King KS, Schaff HV, Stephens EH (2022). Early and Late Outcomes of the Warden and Modified Warden Procedure. *The Annals of Thoracic Surgery*.

[54] Okonta KE, Agarwal V (2012). Does Warden’s procedure reduce sinus node dysfunction after surgery for partial anomalous pulmonary venous connection?. *Interactive Cardiovascular and Thoracic Surgery*.

[55] Kadowaki S, Saprungruang A, Dragulescu A, Yoo SJ, Haller C (2023). Modified Warden Procedure for Partial Anomalous Pulmonary Venous Drainage to Promote Growth. *The Annals of Thoracic Surgery*.

[56] Kumar TKS, Chen D, Halpern D, Bhatla P, Saharan S, Argilla M (2020). Modified Warden operation using aortic homograft. *JTCVS Techniques*.

[57] Danton MHD, Kesieme EB (2023). Modification of the Warden Procedure for Surgical Repair of Partial Anomalous Pulmonary Venous Connection. *World Journal for Pediatric & Congenital Heart Surgery*.

[58] Lenoir M, Rahmani B, Dubois G, Aldebert P, Amrous A, Casalta A (2021). Repair of sinus venosus defects with partial anomalous pulmonary venous connection in adult: Four surgical procedures for a large armamentarium. *Archives of Cardiovascular Diseases Supplements*.

[59] Zubritskiy A, Naberukhin Y, Arkhipov A, Gorbatykh Y, Khapaev T, Nichay N (2020). Outcomes of Double-patch and Warden Techniques in Patients With Supracardiac Partial Anomalous Pulmonary Venous Connection. *Heart, Lung & Circulation*.

[60] Yong MS, Griffiths S, Robertson T, Brink J, d’Udekem Y, Brizard C (2018). Outcomes of the Warden procedure for partial anomalous pulmonary venous drainage in children. *Interactive Cardiovascular and Thoracic Surgery*.

[61] Lin H, Yan J, Wang Q, Li S, Sun H, Zhang Y (2020). Outcomes of the Warden Procedure for Partial Anomalous Pulmonary Venous Drainage. *Pediatric Cardiology*.

[62] Lee ME, Sade RM (1979). Coronary sinus septal defect. Surgical considerations. *The Journal of Thoracic and Cardiovascular Surgery*.

[63] Quaegebeur J, Kirklin JW, Pacifico AD, Bargeron LM (1979). Surgical experience with unroofed coronary sinus. *The Annals of Thoracic Surgery*.

[64] Sugimori H, Nakao T, Ikegaya Y, Iwahashi D, Tsuda S, Kume N (2021). Coronary sinus atrial septal defects in adults over the past 20 years at new Tokyo hospital: case series. *Journal of Cardiothoracic Surgery*.

[65] Ozaki N, Wakita N, Inoue K, Yamada A (2009). Surgical repair of coronary sinus atrial septal defect and supraventricular tachycardia. *Interactive Cardiovascular and Thoracic Surgery*.

[66] Jalal Z, Hascoet S, Baruteau AE, Iriart X, Kreitmann B, Boudjemline Y (2016). Long-term Complications After Transcatheter Atrial Septal Defect Closure: A Review of the Medical Literature. *The Canadian Journal of Cardiology*.

[67] Aruni B, Sharifian A, Eryazici P, Herrera CJ (2013). Late bacterial endocarditis of an Amplatzer atrial septal device. *Indian Heart Journal*.

[68] Amedro P, Soulatges C, Fraisse A (2017). Infective endocarditis after device closure of atrial septal defects: Case report and review of the literature. *Catheterization and Cardiovascular Interventions: Official Journal of the Society for Cardiac Angiography & Interventions*.

[69] Divekar A, Gaamangwe T, Shaikh N, Raabe M, Ducas J (2005). Cardiac perforation after device closure of atrial septal defects with the Amplatzer septal occluder. *Journal of the American College of Cardiology*.

[70] Kamla CE, Buech J, Doldi PM, Hagl C, Juchem G, Dashkevich A (2021). Atrio-aortic erosion caused by Amplatzer Atrial Septal Occluder - a case report. *Journal of Cardiothoracic Surgery*.

[71] Sauer HH, Ntalakoura K, Haun C, Le TP, Hraska V (2006). Early cardiac perforation after atrial septal defect closure with the Amplatzer septal occluder. *The Annals of Thoracic Surgery*.

[72] Konstantinov IE, Kotani Y, Buratto E, Schulz A, Ivanov Y (2022). Minimally invasive approaches to atrial septal defect closure. *JTCVS Techniques*.

[73] Hardman G, Zacharias J (2023). Minimal-Access Atrial Septal Defect (ASD) Closure. *Journal of Cardiovascular Development and Disease*.

[74] Lenoir M, Degirmenci S, Rauzier J, Aldebert P, Casalta A, Gran C (2021). Closure of atrial septal defect by minimally invasive surgery: An alternative approach. *Archives of Cardiovascular Diseases Supplements*.

[75] Yang X, Hu Y, Dong J, Huang P, Luo J, Yang G (2022). Rightvertical axillary incision for atrial septal defect: a propensity score matched study. *Journal of Cardiothoracic Surgery*.

[76] Poyrazoglu HH, Avsar MK, Demir S, Karakaya Z, Güler T, Tor F (2013). Atrial septal defect closure: comparison of vertical axillary minithoracotomy and median sternotomy. *The Korean Journal of Thoracic and Cardiovascular Surgery*.

[77] Yanagisawa J, Maekawa A, Sawaki S, Tokoro M, Ozeki T, Orii M (2019). Three-port totally endoscopic repair vs conventional median sternotomy for atrial septal defect. *Surgery Today*.

[78] Nishida H, Nakatsuka D, Kawano Y, Hiraiwa N, Takanashi S, Tabata M (2017). Outcomes of Totally Endoscopic Atrial Septal Defect Closure Using a Glutaraldehyde-Treated Autologous Pericardial Patch. *Circulation Journal: Official Journal of the Japanese Circulation Society*.

[79] Cheng Y, Chen H, Mohl W, Liu X, Si Z (2013). Totally endoscopic congenital heart surgery compared with the traditional heart operation in children. *Wiener Klinische Wochenschrift*.

[80] Ma ZS, Yin QY, Dong MF, Feng ZY, Wang LX (2011). Quality of life in patients undergoing totally thoracoscopic closure for atrial septal defect. *The Annals of Thoracic Surgery*.

